# Dental Procedure-Related Emphysema: A Scoping Review of Clinical Presentation, Anatomical Distribution, Management, and Outcomes

**DOI:** 10.3390/dj14090602

**Published:** 2026-09-17

**Authors:** Ulrike Kuchler, Laura Scholter, Reinhard Gruber

**Affiliations:** 1 Department of Oral Surgery, University Clinic of Dentistry, Medical University of Vienna, Sensengasse 2a, 1090 Vienna, Austria; n11700926@students.meduniwien.ac.at; 2 Department of Oral Biology, University Clinic of Dentistry, Medical University of Vienna, Sensengasse 2a, 1090 Vienna, Austria; 3 Austrian Cluster for Tissue Regeneration, 1200 Vienna, Austria; 4 Department of Periodontology, School of Dental Medicine, University of Bern, Freiburgstrasse 7, 3010 Bern, Switzerland

**Keywords:** subcutaneous emphysema, pneumomediastinum, dentistry, oral surgery, air polishing, computed tomography, scoping review

## Abstract

**Background/Objectives:** Dental procedure-related emphysema is an infrequently reported complication of dental care that may extend from superficial facial tissues into deep cervical, mediastinal, orbital, thoracic, intracranial, or vascular compartments. This revised scoping review aimed to map contemporary published cases using an expanded multi-database search and to characterize procedures, anatomical distribution, clinical presentation, diagnostic assessment, management, complications, and outcomes. **Methods:** PubMed/MEDLINE, Scopus, and Web of Science Core Collection were searched for English-language reports published from 1 January 2016 through 26 August 2026. Records were deduplicated and screened using predefined eligibility criteria; data were charted descriptively in accordance with PRISMA-ScR. **Results:** The expanded searches yielded 642 database records. After application of the publication window and deduplication, 246 unique records were screened. The synthesis included 109 studies describing 129 patients. The case literature remained dominated by extraction-related events, while restorative/prosthodontic treatment, dental cleaning/air-polishing, and endodontic procedures were also represented. Facial and cervical emphysema remained the most frequently reported distributions, and mediastinal and deep fascial-space extension were prominent among published cases. CT and antibiotics were frequently reported, but the case-report evidence does not establish their comparative effectiveness. **Conclusions:** Dental procedure-related emphysema is represented by a heterogeneous case literature with strong potential for publication bias. Sudden swelling with crepitus after dental treatment should prompt immediate assessment, with imaging and escalation guided by suspected anatomical extent and clinical severity. Reported frequencies should not be interpreted as incidence, procedure-specific risk, or evidence of treatment effectiveness.

## 1. Introduction

Dental procedure-related emphysema is an infrequently reported but clinically important iatrogenic complication caused by the introduction of pressurized air or gas into facial or cervical soft tissues. Air may remain localized or dissect along connected fascial planes into the parapharyngeal, retropharyngeal, mediastinal, orbital, thoracic, intracranial, or vascular compartments. Rare published complications include pneumothorax, airway compromise, visual or neurologic involvement, air embolism, cardiovascular collapse, and death. Sudden swelling during or shortly after dental treatment, particularly when accompanied by palpable crepitus, is therefore an important diagnostic clue [1,2,3].

The anatomical pattern of spread is determined by the point of entry and the continuity of the cervicofacial spaces. Air introduced through an extraction socket, periodontal or peri-implant pocket, root canal, mucosal laceration, or operative wound can enter the buccal, sublingual, submandibular, masticator, parapharyngeal, and retropharyngeal spaces. Posterior cervical pathways may communicate with the mediastinum, while maxillary and infratemporal routes can extend toward the pterygopalatine region and orbit. The resulting presentation may therefore range from isolated facial swelling to dysphagia, dysphonia, chest discomfort, dyspnea, orbital symptoms, or neurologic deterioration [1,2,3].

Dental emphysema has been described after tooth extraction, restorative and endodontic treatment, periodontal therapy, implant procedures, sinus-related dental surgery, and professional dental cleaning or air-polishing. High-speed air turbines, air–water syringes, air-driven handpieces, and subgingival air-polishing systems are frequently implicated, although cases can occur without a forward-venting air instrument when postoperative pressure-generating behaviors or other routes of air entry are present [1,2,3,4].

Jones et al. systematically reviewed 135 cases reported from 1993 to 2020 and characterized dental causes, anatomical locations, implicated devices, and management [1]. Subsequent reviews have focused on mediastinal extension, cervicofacial emphysema, and endodontic mechanisms [2,3,4]. The present scoping review was designed as a contemporary extension that maps cases from 2016 onward and integrates patient-level information on clinical presentation, anatomical extent, diagnostic investigations, management, complications, and outcomes. The scoping design was selected because the evidence is heterogeneous and predominantly descriptive; the objective is evidence mapping rather than estimation of incidence, comparative risk, or treatment effects.

## 2. Materials and Methods

### 2.1. Study Design and Reporting

This scoping review was conducted and reported in accordance with the Preferred Reporting Items for Systematic Reviews and Meta-Analyses Extension for Scoping Reviews (PRISMA-ScR) [5]. The protocol was not prospectively registered. The review was designed to map the contemporary case literature, characterize recurring clinical patterns, and identify evidence gaps rather than to estimate incidence, procedure-specific risk, or treatment effectiveness.

### 2.2. Information Sources and Search Strategy

PubMed/MEDLINE, Scopus, and Web of Science Core Collection were searched for reports published from 1 January 2016 through 26 August 2026. The PubMed strategy combined terms for subcutaneous, cervicofacial, mediastinal, surgical, and orbital emphysema with dental, oral-surgical, extraction, endodontic, periodontal, implant, restorative, air-polishing, air-turbine, and high-speed handpiece terms in title/abstract fields. Equivalent topic searches were adapted for Scopus (TITLE-ABS-KEY) and Web of Science Core Collection (TS). No case-report publication-type filter was applied because inconsistent indexing could reduce sensitivity. The complete reproducible search strings and database-specific processing are provided in Supplementary Table S1. Reference lists of relevant reviews and included reports were checked for eligible records.

### 2.3. Eligibility Criteria

Dental procedure-related emphysema was operationally defined as clinically or radiologically demonstrated air in facial, cervical, mediastinal, orbital, thoracic, intracranial, vascular, intraosseous, pericardial, or named deep fascial compartments occurring during or after dental or dental-related care. Only English-language literature was eligible. Case reports and case series were included when sufficient patient-level information was available to establish the dental exposure and clinical diagnosis/course. Dental-related procedures included oral surgery, implant treatment, endodontic and periodontal procedures, restorative/prosthodontic treatment, and dental cleaning or air-polishing. Reviews without new patients, editorials, conference abstracts without a full report, experimental studies, duplicate publications of the same patient, non-English full texts, and reports unrelated to dental care were excluded.

### 2.4. Record Management and Study Selection

The three database exports were combined and processed against the prespecified publication window. PubMed yielded 198 records, Scopus 99, and Web of Science 345 (642 database records in total). The exported Scopus and Web of Science sets contained records outside the review period; 273 records were removed on application of the 2016–2026 window. A further 123 duplicate or overlapping database records were removed, leaving 246 unique records for title/abstract screening. Reports considered potentially eligible underwent full-text/source assessment. Duplicate publications and potential reports of the same patient were compared using DOI, title, authorship, institution, age/sex, procedure, treated tooth, and clinical chronology. One investigator (L.S.) screened titles and abstracts and assessed potentially eligible reports against the predefined criteria. Uncertain eligibility decisions and potential duplicate publications were reviewed with the coauthors and resolved by consensus. The same eligibility assessment was applied to reports identified from the previous review dataset or by supplementary source checking.

### 2.5. Data Charting

One investigator (L.S.) charted patient-level data in a standardized spreadsheet using predefined variables. Variables included age, sex, relevant medical history, procedure, jaw and tooth group, documented or possible air source, self-induced pressure-generating mechanisms, anatomical distribution, swelling, crepitus, pain, respiratory or upper aerodigestive symptoms, imaging, antibiotic use, complications, follow-up, complete resolution time, and persistent morbidity or death. Missing or unavailable information was coded as not reported. Procedure categories were coded as multiple-response variables; when more than one relevant procedure was reported for an individual patient, all applicable categories were retained. Ambiguous source descriptions were discussed among the authors and resolved by consensus. Generative AI was used only to assist with administrative organization of screening records, standardization of tabular material, flagging possible duplicate or internally inconsistent entries, arithmetic checks of summary counts, and preparation of graphical materials. It did not make final eligibility decisions or independently determine patient-level data. Final inclusion/exclusion decisions, patient-level data charting/extraction, and verification of the values used in the synthesis were performed by the authors; all AI-assisted checks and summaries were reviewed and, where necessary, corrected by the authors.

### 2.6. Anatomical Classification

Because source terminology was heterogeneous, an author-developed descriptive classification was used. Facial involvement included subcutaneous air in cheek, buccal, perioral, temporal, infraorbital, or other facial soft tissues; cervical involvement comprised subcutaneous air in the neck; and deep fascial-space involvement comprised named spaces such as submandibular, sublingual, masticator, parapharyngeal, retropharyngeal, infratemporal, or pterygopalatine spaces. Mediastinal, orbital, thoracic subcutaneous, intracranial, vascular, intraosseous, pericardial, and scalp categories followed the source descriptions. Individual patients could be assigned to more than one anatomical category.

### 2.7. Data Synthesis and Critical Appraisal

Data were synthesized descriptively. Patient-level frequencies and percentages use the denominator of 129 patients unless otherwise specified, and multiple-response categories may therefore sum to more than 100%. Resolution time was summarized at the episode level using the numerical time to complete resolution. No meta-analysis was performed because the evidence consisted of heterogeneous noncomparative case reports and case series. Consistent with the mapping purpose of this scoping review and the predominantly descriptive case-report evidence, no formal risk-of-bias assessment was undertaken. Frequencies were interpreted as patterns in the published literature rather than incidence or risk estimates.

## 3. Results

### 3.1. Study Selection

**Figure 1:**
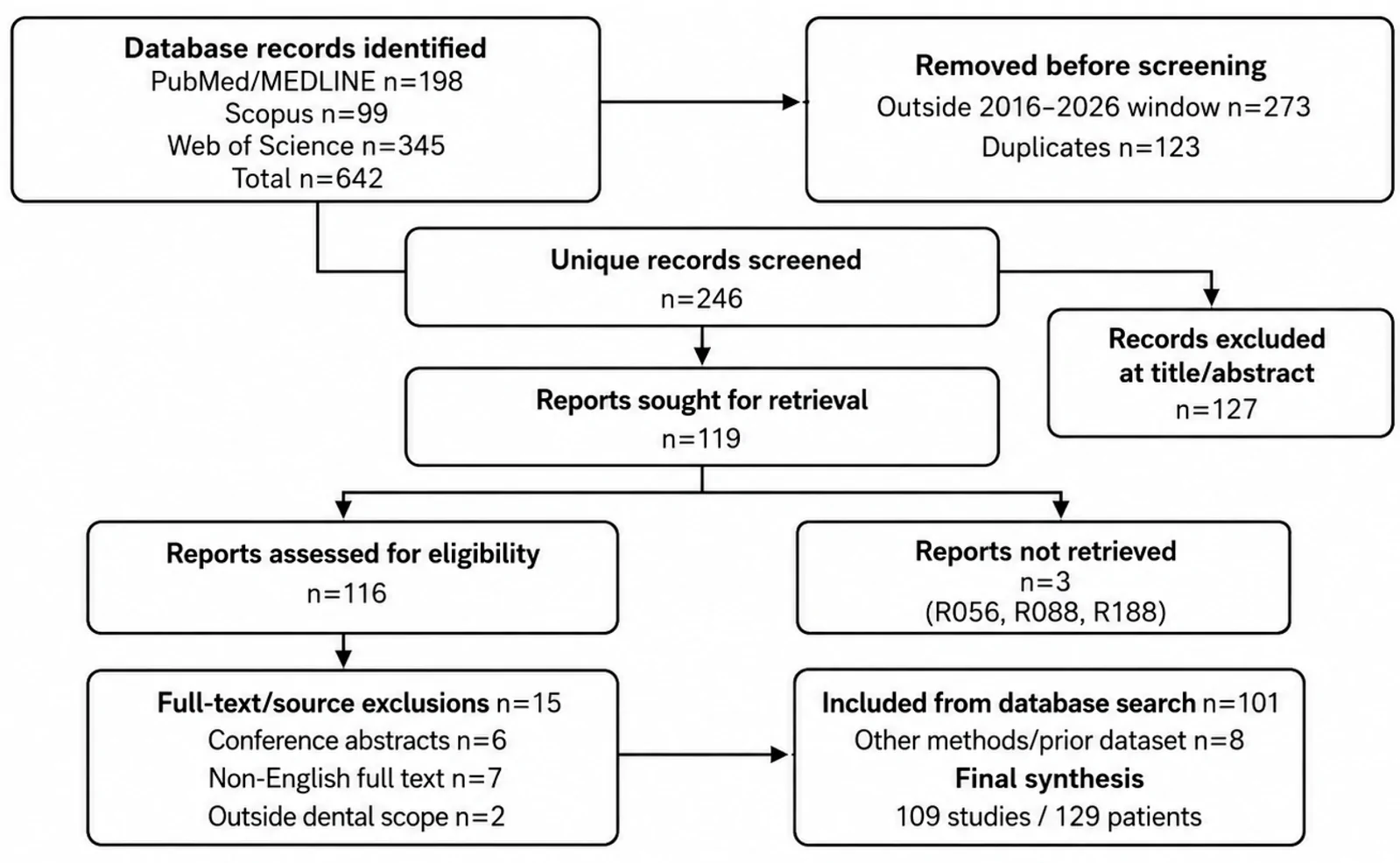
Study selection after the expanded multi-database search. The searches yielded 642 records; after date restriction and deduplication, 246 unique records were screened. Of 119 reports sought, 116 were retrieved and 101 met the eligibility criteria; eight additional eligible reports were identified through the previous dataset or supplementary source checking. The final synthesis included 109 studies describing 129 patients.

The expanded database searches identified 642 records (PubMed/MEDLINE, n = 198; Scopus, n = 99; Web of Science Core Collection, n = 345). Application of the prespecified 2016–2026 publication window removed 273 records, and deduplication removed 123 additional records, leaving 246 unique records for screening. Title/abstract screening excluded 127 records, and 119 reports were sought for retrieval. Three reports could not be retrieved. Of 116 reports assessed for eligibility, 15 were excluded at the source/full-text stage (6 conference abstracts, 7 non-English full texts, and 2 reports outside the predefined dental-procedure scope). One hundred and one eligible reports arose from the expanded database corpus. Eight additional eligible reports were retained from the previous review dataset or identified by supplementary source checking, resulting in a final synthesis of 109 studies describing 129 patients (Figure 1; Supplementary Tables S1 and S4) [5].

### 3.2. Patient and Study Characteristics

The published sample comprised 129 patients. Female sex was reported for 74/129 patients (57.4%), male sex for 47/129 (36.4%), and sex was not reported for 8 patients (6.2%). Reported ages ranged from 2.5 to 84 years. Included publications originated from multiple clinical disciplines and settings, including oral and maxillofacial surgery, restorative dentistry, endodontics, periodontology, implant dentistry, emergency medicine, otolaryngology, ophthalmology, and anesthesiology. The 109 included studies are listed in Supplementary Table S2 and are cited in the reference list [2,6,7,8,9,10,11,12,13,14,15,16,17,18,19,20,21,22,23,24,25,26,27,28,29,30,31,32,33,34,35,36,37,38,39,40,41,42,43,44,45,46,47,48,49,50,51,52,53,54,55,56,57,58,59,60,61,62,63,64,65,66,67,68,69,70,71,72,73,74,75,76,77,78,79,80,81,82,83,84,85,86,87,88,89,90,91,92,93,94,95,96,97,98,99,100,101,102,103,104,105,106,107,108,109,110,111,112,113].

### 3.3. Associated Procedures and Tooth Distribution

**Figure 2:**
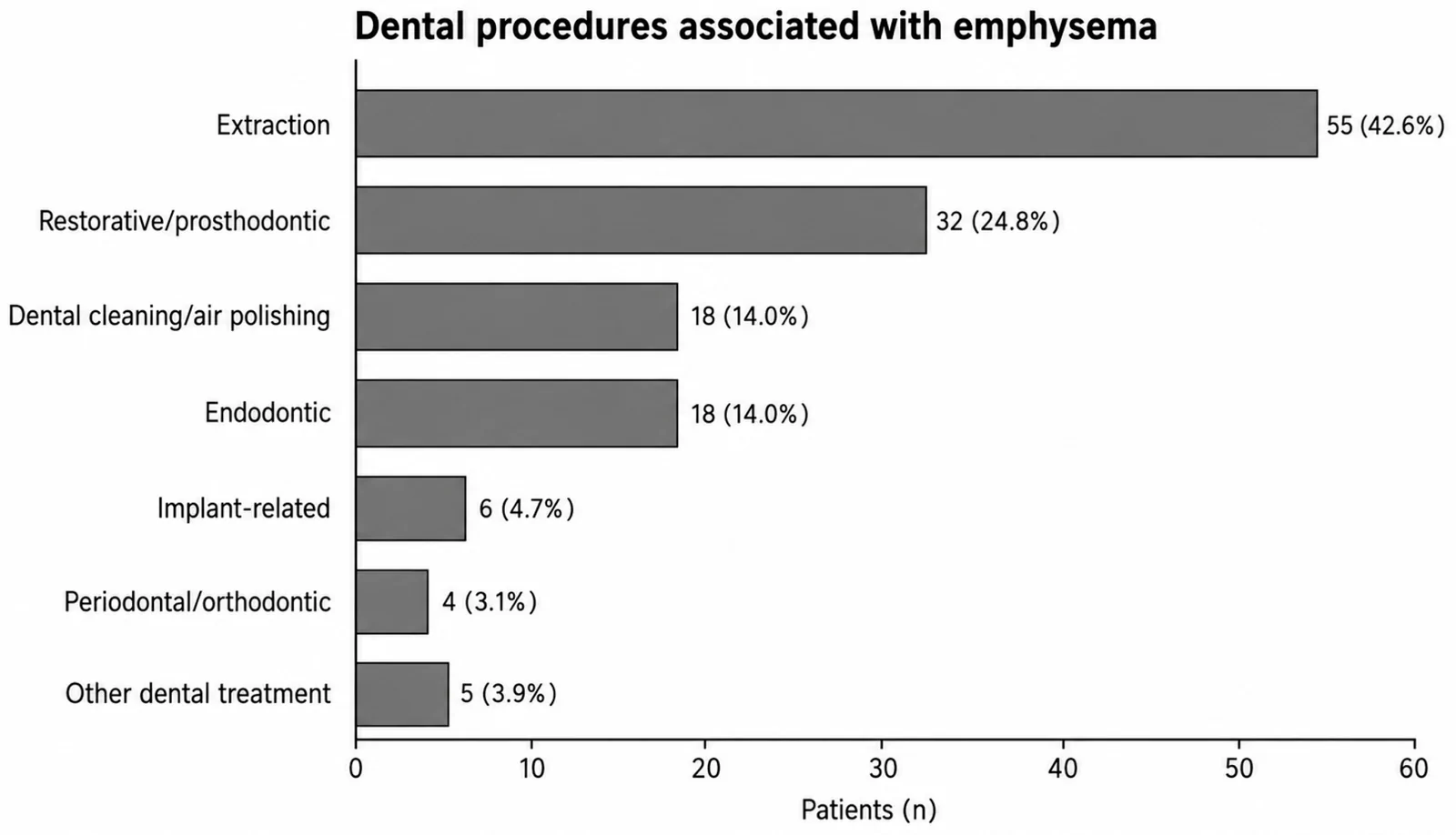
Dental procedures associated with dental procedure-related emphysema. Horizontal bars show the number and percentage of patients according to the dental procedure temporally associated with the development of emphysema. Tooth extraction was the most frequently represented procedure, followed by restorative/prosthodontic treatment, dental cleaning or air-polishing, and endodontic treatment. Implant-related, periodontal/orthodontic, and other dental procedures were reported less frequently. Percentages are calculated using the total cohort of 129 patients and therefore should be interpreted as frequencies within the published case literature rather than estimates of procedure-specific incidence or risk.

**Figure 3:**
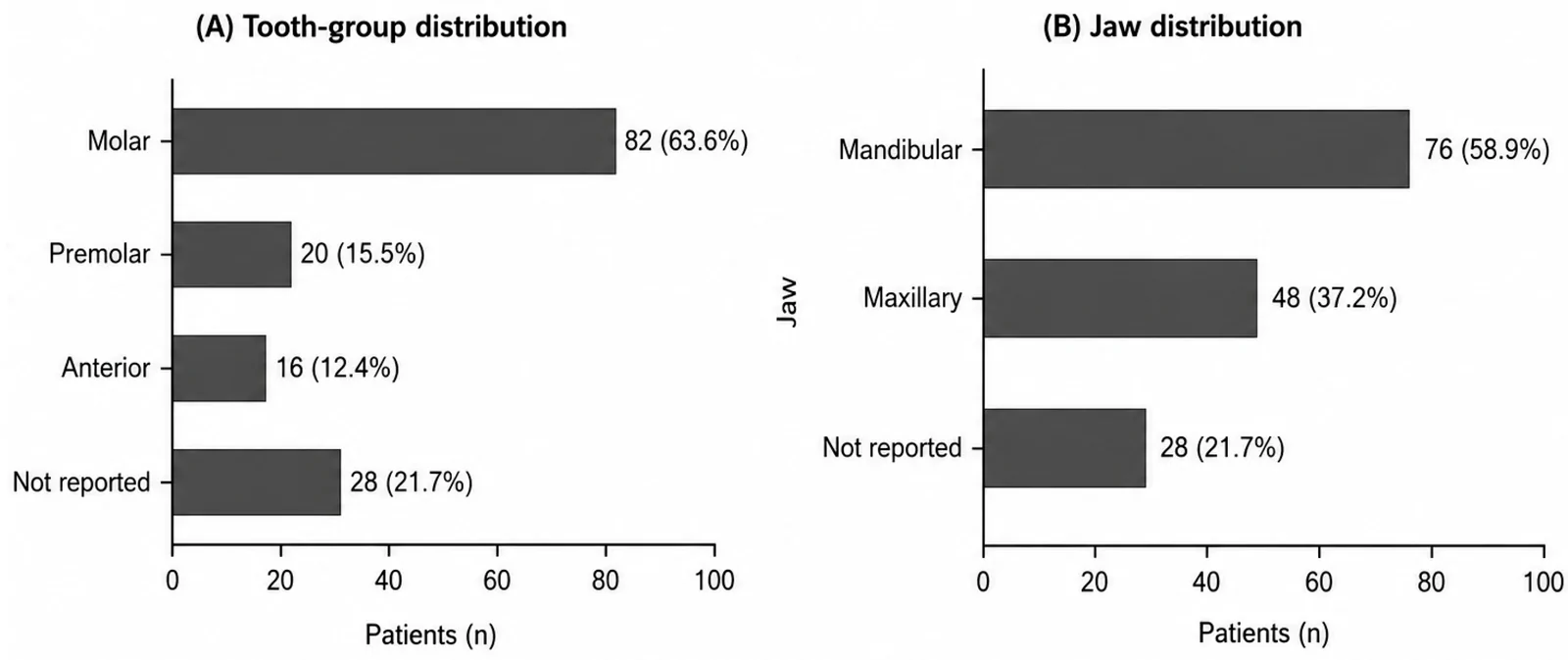
Tooth group and jaw distribution of dental procedures associated with emphysema. (**A**) Horizontal bars show the number and percentage of patients according to the tooth group involved in the reported dental procedure. Molars were the most frequently involved tooth group, followed by premolars and anterior teeth. The category “Not reported” indicates that the affected tooth group was not reported in the source publication. More than one tooth group could be involved in an individual patient. (**B**) Horizontal bars show the number and percentage of patients according to jaw location (mandible, maxilla, or not reported). Mandibular procedures were represented more frequently than maxillary procedures in the published case literature. Not reported indicates that the jaw location was not clearly specified in the original report. Percentages in both panels are calculated using the total cohort of 129 patients. Because categories were not always mutually exclusive and because of missing reporting in some studies, percentages should be interpreted as descriptive frequencies within the published case literature rather than as epidemiologic estimates.

Figure 2 shows that extraction remained the most frequently represented procedure category (55/129, 42.6%), followed by restorative/prosthodontic treatment (32/129, 24.8%), dental cleaning or air-polishing (18/129, 14.0%), endodontic treatment (18/129, 14.0%), implant-related procedures (6/129, 4.7%), and periodontal/orthodontic or other dental procedures. Mandibular involvement was reported in 76 patients (58.9%) and maxillary involvement in 48 (37.2%). Molars were the most frequently represented tooth group (82/129, 63.6%; Figure 3). These frequencies describe the published cases and should not be interpreted as procedure-, jaw-, or tooth-specific risk [6,18,20,21,24,29,30,44,59,75,85,104,108].

### 3.4. Anatomical Distribution and Clinical Presentation

**Figure 4:**
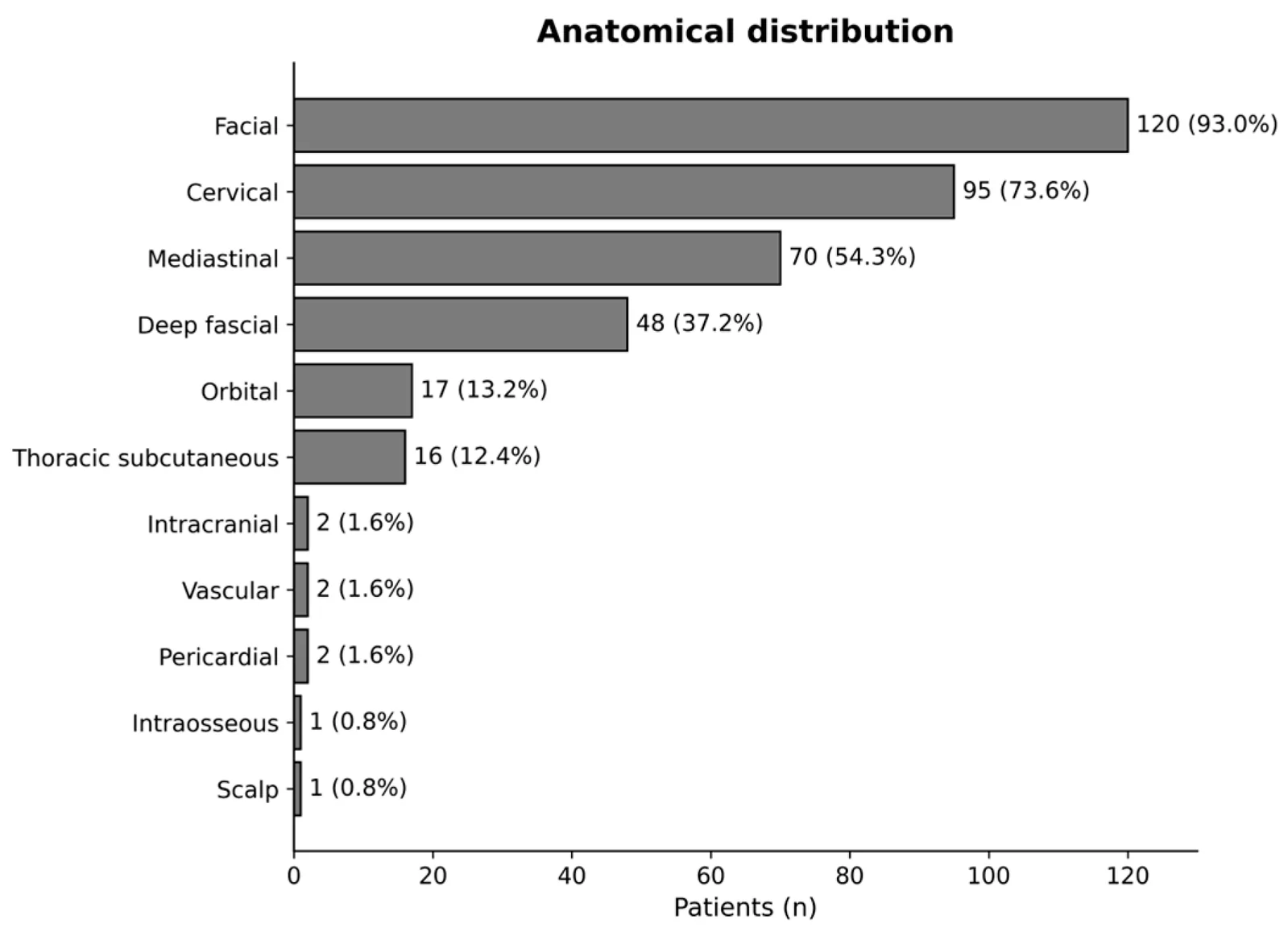
Anatomical distribution of dental procedure-related emphysema. Horizontal bars show the number and percentage of patients in whom air was reported within each anatomical region. Facial subcutaneous emphysema was the most frequently reported distribution, followed by cervical involvement, mediastinal emphysema, and involvement of named deep fascial spaces. Less frequent anatomical extensions included the orbit, thoracic subcutaneous tissues, intracranial compartment, vascular system, pericardium, intraosseous sites, and scalp. Anatomical categories were derived from the terminology used in the individual source reports and represent an author-developed descriptive classification. Because air frequently extended across several connected anatomical compartments, individual patients could be assigned to multiple categories. Percentages are based on 129 patients and therefore do not sum to 100%.

**Figure 5:**
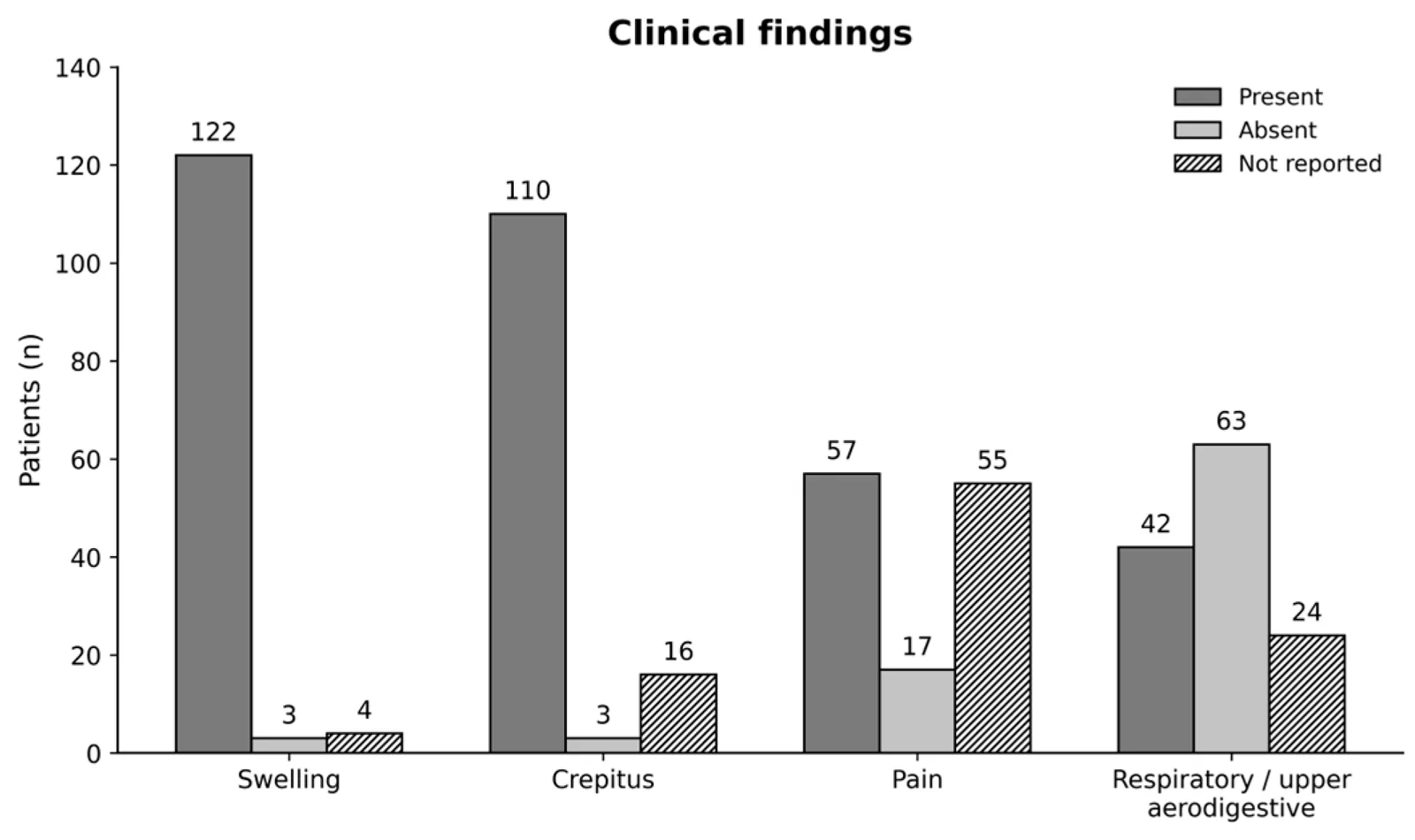
Clinical findings reported in patients with dental procedure-related emphysema. Grouped bars show whether swelling, palpable crepitus, pain, and respiratory or upper aerodigestive symptoms were reported as present, absent, or not reported. Swelling and palpable crepitus were the most consistently documented clinical findings. Pain and respiratory or upper aerodigestive symptoms were less uniformly reported and showed a larger proportion of missing information. “Not reported” indicates that the respective finding could not be determined from the source publication. Values represent patient counts within the total cohort of 129 patients.

Figure 4 summarizes that facial subcutaneous emphysema was reported in 120 patients (93.0%), cervical emphysema in 95 (73.6%), mediastinal emphysema in 70 (54.3%), and named deep fascial-space involvement in 48 (37.2%). Orbital involvement was reported in 17 patients (13.2%). Less frequent reported compartments included thoracic subcutaneous tissues, intracranial or spinal/extradural spaces, the vascular system, pericardium, intraosseous sites, and scalp. Clinical findings reported swelling in 122 patients, crepitus in 110, pain in 57, and respiratory or upper aerodigestive symptoms in 42 (Figure 5) [7,11,31,32,33,35,39,42,73,77,90,102,105].

### 3.5. Diagnostic Assessment and Implicated Mechanisms

**Figure 6:**
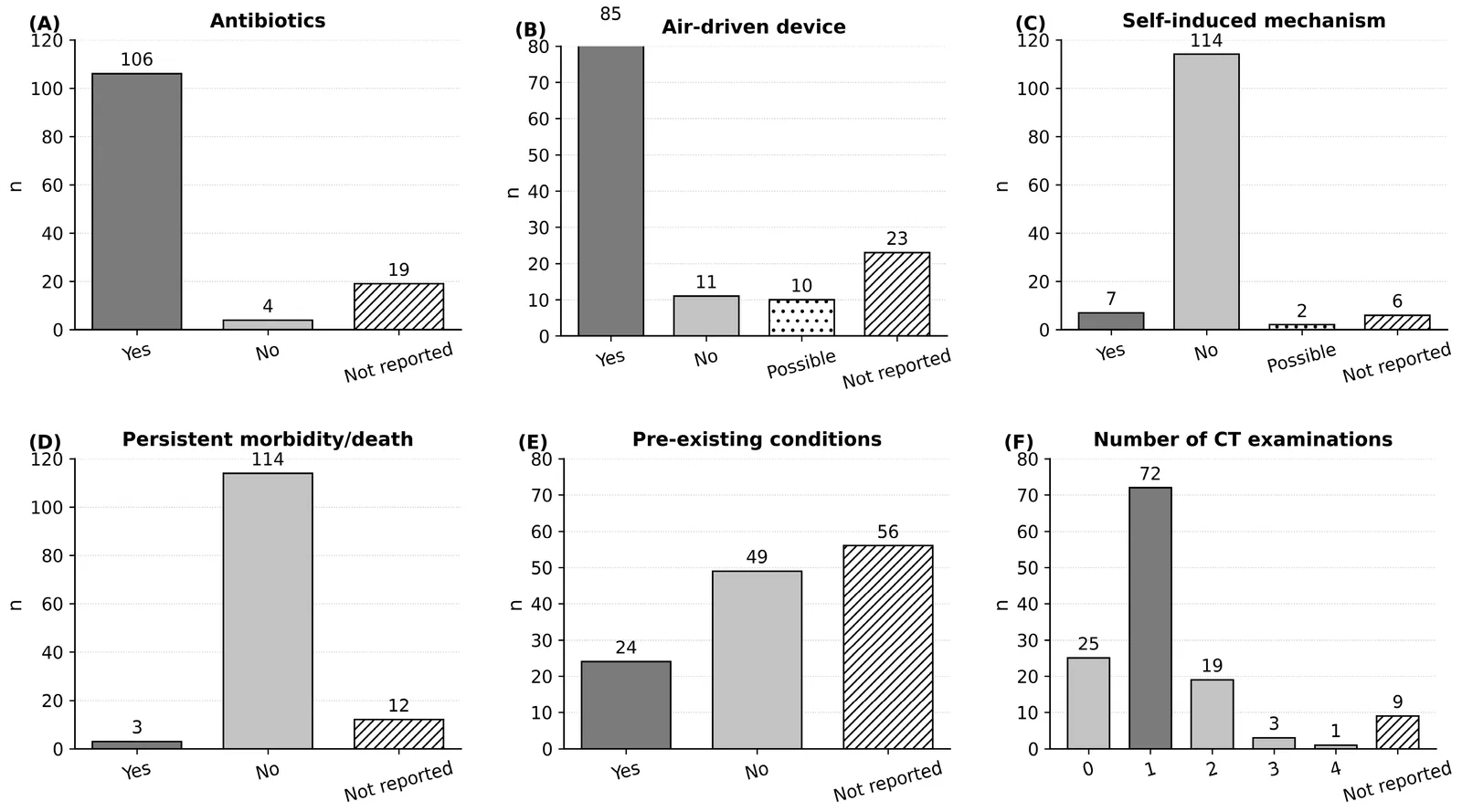
(**A**–**F**) Diagnostic assessment, management, implicated mechanisms, and outcomes in patients with dental procedure-related emphysema. The six panels summarize selected patient-level variables across the 129 included patients: antibiotic use (**A**), documented use of an air-driven device (**B**), self-induced or pressure-generating (**C**) mechanisms, persistent morbidity (**D**) or death, pre-existing medical conditions (**E**), and the number of computed tomography (CT) examinations (**F**). Categories distinguish explicitly documented findings from absent, possible, or unreported information where applicable. Antibiotic administration and use of an air-driven device were frequently reported (**A**,**B**), whereas self-induced mechanisms and persistent morbidity or death were uncommon (**C**). CT was the principal cross-sectional imaging modality used to establish anatomical extent and to evaluate deep cervical, mediastinal, thoracic, orbital, or other complications (**F**). These frequencies describe management and reporting patterns within published cases and should not be interpreted as evidence of treatment effectiveness or diagnostic necessity.

CT was frequently used to confirm diagnosis and delineate anatomical extent (Figure 6). Overall, 95/129 patients (73.6%) underwent at least one CT examination: 72 underwent one CT examination, 19 underwent two, 3 underwent three, and 1 underwent four. Twenty-five patients were coded as having no CT examination, and CT frequency was not reported for 9. An air-driven device was explicitly documented in 85 patients, explicitly absent in 11, considered possible in 10, and not reported in 23. Pressure-generating patient behavior was documented in 7 cases and considered possible in 2. These variables distinguish documented mechanisms from plausible or inferred routes of air entry [2,12,16,35,75,80,85,103,104,108].

### 3.6. Management and Outcomes

**Figure 7:**
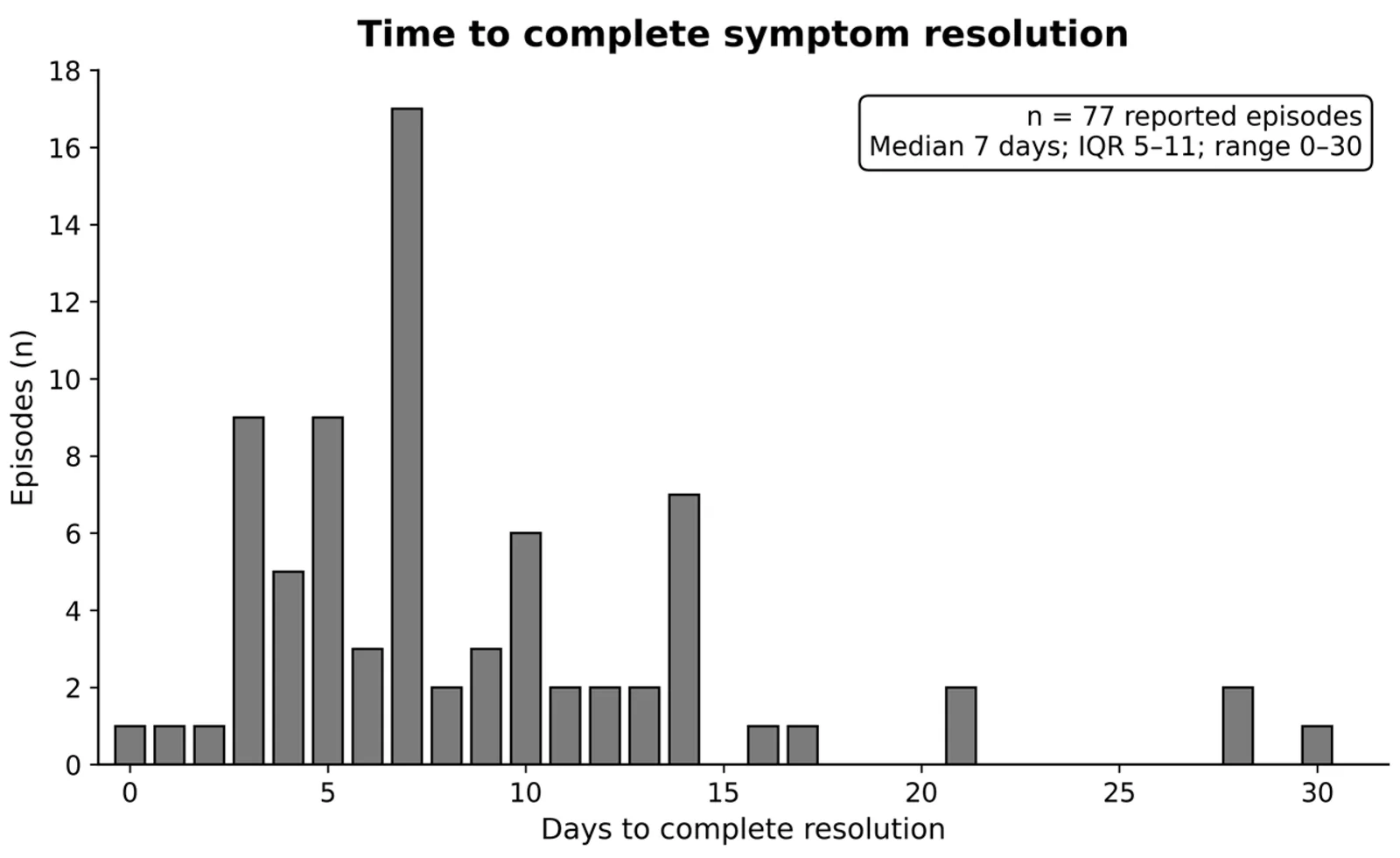
Time to complete symptom resolution in reported episodes of dental procedure-related emphysema. Bars show the frequency distribution of the reported time from onset to complete clinical resolution among 77 episodes for which a numerical resolution time was available. The median time to complete resolution was 7 days, with an interquartile range of 5–11 days and an observed range of 0–30 days. Resolution time is presented at the episode level because individual patients could experience more than one temporally distinct episode. Cases without a numerical resolution time were excluded from this distribution. The figure describes the course of published cases and should not be interpreted as a population-based estimate of recovery time.

Antibiotic therapy was reported in 106 patients (82.2%), was explicitly not used in 4, and was not reported in 19. Reported management also included observation, analgesia, oxygen, avoidance of pressure-increasing activities, hospital monitoring, specialist consultation, and, in selected severe cases, invasive airway/thoracic management or hyperbaric oxygen. Persistent morbidity or death was documented in 3 patients; no persistent morbidity was reported in 114, while long-term status was not reported in 12 (Figure 6). Across 77 episodes with an exact reported time to complete resolution, the median was 7 days (interquartile range, 5–11 days; range, 0–30 days) (Figure 7). These descriptive management frequencies cannot establish the effectiveness of antibiotics, oxygen, imaging, hospital admission, or conservative care [1,2,3,4,6,7,8,9,90,102].

## 4. Discussion

### 4.1. Principal Findings

This expanded scoping review maps 109 studies describing 129 patients with dental procedure-related emphysema published from 2016 through August 2026. Broadening the information sources from PubMed alone to PubMed/MEDLINE, Scopus, and Web of Science substantially increased the screened evidence base and identified additional case reports and case series that were not captured by the original search. Extraction remained the most frequently represented context in the published literature, but restorative/prosthodontic procedures, dental cleaning or air-polishing, endodontic treatment, implant therapy, and other procedures were also represented. Facial and cervical involvement predominated, while mediastinal and deep fascial-space extension remained prominent among published cases. The expanded corpus also contains rare orbital, intracranial, vascular, pericardial, and thoracic complications.

### 4.2. Added Value Relative to Previous Reviews

The present review complements rather than replaces earlier historical syntheses. Jones et al. examined 135 cases reported from 1993 to 2020 and focused on causes, anatomical location, devices, and management [1]. The current scoping review uses a contemporary 2016–2026 window, an expanded multi-database search, and a broader patient-level charting framework covering presenting symptoms, detailed anatomical spread, diagnostic investigations, management patterns, complications, resolution time, and persistent outcomes. The new search also captures recently reported air-polishing, peri-implant, orbital, neurologic, and air-embolism presentations that post-date or were outside earlier evidence syntheses.

### 4.3. Publication Bias and Interpretation of Frequencies

The published sample is strongly susceptible to publication and selection bias. Extensive, unusual, or life-threatening events are more likely to be written up than mild episodes that resolve rapidly in routine dental practice. Database indexing, English-language restriction, and inconsistent terminology also influence retrieval. Consequently, the observed frequencies of mediastinal extension, CT use, antibiotic treatment, or particular procedures are properties of the published case literature, not estimates of incidence, comparative risk, or typical clinical distribution.

### 4.4. Mechanisms and Anatomical Spread

The reported anatomical patterns are consistent with the continuity of cervicofacial fascial spaces, but the mechanism of air entry was not always directly documented. A tissue defect together with a pressurized air source is a recurring pattern: extraction sockets, periodontal or peri-implant pockets, root canals, crestal perforations, gingival lacerations, and surgical wounds can provide access to loose connective-tissue planes. Air may pass through submandibular, sublingual, masticator, parapharyngeal, or retropharyngeal spaces toward the mediastinum, while maxillary/infratemporal pathways can reach the orbit [1,2,3,4]. Several recent cases additionally show that electric handpieces, postoperative emesis, nose blowing, or procedures without a classic forward-venting turbine can still be associated with emphysema [12,85,103]. These observations support distinguishing directly documented devices/actions from inferred anatomical routes.

### 4.5. Clinical Recognition and Imaging

Sudden swelling during or shortly after dental care should prompt palpation for crepitus, review of the procedure and instruments used, assessment of vital signs, and attention to airway, swallowing, voice, chest, ocular, and neurologic symptoms. Differential diagnoses include allergic reaction, angioedema, hematoma, infection, postoperative edema, pneumoparotitis, and necrotizing fasciitis. CT is particularly informative when symptoms extend beyond a localized superficial area, when cervical, mediastinal, orbital, thoracic, or neurologic involvement is suspected, or when the diagnosis is uncertain [1,2,3,4]. However, case reports cannot establish that every clinically stable localized case requires CT or repeated imaging; imaging should be individualized to the suspected anatomical extent and clinical severity.

### 4.6. Management and Escalation of Care

Antibiotics were frequently administered in published cases, but the available evidence cannot demonstrate prophylactic benefit or define evidence-based indications. The same limitation applies to oxygen therapy, hospital admission, serial CT, corticosteroids, and other interventions. Clinical escalation is nevertheless warranted when airway stability is uncertain, symptoms are progressive, deep-space or mediastinal spread is suspected, systemic inflammatory findings occur, or orbital, neurologic, thoracic, or vascular complications are present. Reports of tension pneumothorax, air embolism, neurologic compromise, and death show why severe presentations require multidisciplinary emergency assessment [6,8,9,90,102].

### 4.7. Prevention and Clinical Implications

The case patterns support plausible clinical precautions rather than comparative evidence-based preventive recommendations. These include avoiding direct pressurized air near extraction sockets, periodontal/peri-implant defects, root canals with compromised apical barriers, mucosal lacerations, and fresh surgical wounds; using non-air-driven or appropriately vented systems when feasible; and adhering to device-specific instructions for subgingival air-polishing [1,2,3,4,10,104,108]. Patients should be advised to avoid forceful nose blowing or other pressure-generating behaviors when a tissue or sinus communication is possible. These implications are biologically plausible but have not been tested in comparative prevention studies.

### 4.8. Strengths and Limitations

A major strength of the revision is the expanded search across three major bibliographic databases with a documented deduplicated screening corpus of 246 unique records. The review also provides structured patient-level mapping across procedures, anatomical sites, clinical findings, investigations, management, and outcomes. Limitations remain substantial. The evidence is almost entirely case reports and small case series; mild events are likely underreported; clinical variables are incompletely and inconsistently described; and causal attribution is uncertain when several instruments or behaviors are plausible. The English-language restriction may exclude relevant non-English cases. The 2016–2026 window intentionally prioritizes contemporary practice but excludes older evidence. Three potentially relevant reports could not be retrieved. Finally, the anatomical categories are author-developed descriptive groupings. Screening and data charting were performed by one investigator, with coauthor discussion of uncertain cases, which may have increased the risk of selection or extraction error.

### 4.9. Future Research

Multicenter registries or structured surveillance systems are needed to estimate incidence and procedure-specific risk using denominators for the number and type of dental procedures performed. Standardized case reporting should document the exact instrument and air source, route of tissue entry, timing of onset, anatomical extent, airway/respiratory/ocular/neurologic findings, rationale for imaging, management decisions, microbiology when relevant, and short- and long-term outcomes. Comparative studies are needed before firm recommendations can be made regarding prophylactic antibiotics, imaging thresholds, oxygen therapy, or admission criteria.

## 5. Conclusions

Dental procedure-related emphysema is represented by a heterogeneous and publication-biased case literature and the present review cannot estimate its incidence. Published cases show that air introduced during or after dental care can remain localized or extend into deep cervical, mediastinal, orbital, thoracic, intracranial, or vascular compartments. Sudden swelling with palpable crepitus should prompt immediate clinical assessment and cessation of pressurized-air use. CT may be considered when deeper extension or complications are suspected, while progressive symptoms, airway concern, systemic illness, orbital or neurologic findings, thoracic complications, or suspected air embolism require urgent escalation. Preventive measures should be understood as clinically plausible precautions derived from recurring case patterns rather than proven interventions.

## Data Availability

All data generated or analyzed in this scoping review are included in the article and its Supplementary Materials. The complete database search strategies and search log, study-level inclusion list, patient-level charted dataset, and full-text/source exclusions and reports not retrieved are provided in Supplementary Tables S1–S4.

## Supplementary Materials

The following supporting information can be downloaded at: https://www.mdpi.com/article/10.3390/dj14090602/s1, Table S1: Complete database search strategies and search log; Table S2: Study-level inclusion list; Table S3: Patient-level charted dataset; Table S4: Full-text/source exclusions and reports not retrieved [114,115,116,117,118,119,120,121,122,123,124,125,126,127,128,129,130,131]; completed PRISMA-ScR checklist.

## Abbreviations

CT, computed tomography; PRISMA-ScR, Preferred Reporting Items for Systematic Reviews and Meta-Analyses Extension for Scoping Reviews; SE, subcutaneous emphysema.
