# Supplementary material for: Dental Procedure-Related Emphysema: A Scoping Review of Clinical Presentation, Anatomical Distribution, Management, and Outcomes
Source: Dent J (Basel). 2026 Sep 17;14(9):602. doi: 10.3390/dj14090602 (PMC13605442; doi:10.3390/dj14090602)
Supplement: Supplementary file 1 [file dentistry-14-00602-s001.zip › dentistry-4515840-PRISMA_ScR_Checklist_PROOF_Modified-0916.pdf]

Completed PRISMA-ScR Reporting Checklist

Dental Procedure-Related Emphysema: A Scoping Review of Clinical Presentation, Anatomical Distribution, Management, and Outcomes

Prepared for revised manuscript V11

Checklist basis: PRISMA Extension for Scoping Reviews (PRISMA-ScR), Tricco et al., Ann Intern Med. 2018;169:467-473. Items 12 and 16 are optional in PRISMA-ScR. “Reported” indicates that the item is addressed in the manuscript/supplement; “N/A” indicates an optional activity not undertaken; “Partial” indicates incomplete reporting of the full item.

Item	Topic	Status	Where reported	Completion note
1	Title	Reported	Title, page 1	The title identifies the work as a scoping review.
2	Structured summary	Reported	Abstract, page 1	Structured Background/Objectives, Methods, Results, and Conclusions summarize the review.
3	Rationale	Reported	Introduction, pages 1-2	Rationale and need for contemporary evidence mapping are explained in relation to prior reviews and heterogeneous case evidence.
4	Objectives	Reported	Abstract; Introduction; Section 2.1	Objectives are stated as mapping contemporary published cases and characterizing procedures, anatomy, presentation, diagnostics, management, complications, and outcomes.
5	Protocol and registration	Reported (none)	Section 2.1, page 2	States that the protocol was not prospectively registered.
6	Eligibility criteria	Reported	Section 2.3, page 3	Operational definition, eligible designs/procedures, language/date limits, and exclusion criteria are specified.
7	Information sources	Reported	Section 2.2; Supplementary Tables S1-S2	PubMed/MEDLINE, Scopus, and Web of Science Core Collection were searched through 26 August 2026. The previous review dataset and supplementary source checking are described, and the eight supplementary-route reports are identified in S2.
8	Search	Reported	Section 2.2; Supplementary Table S1	Search concepts/fields described in text; complete database-specific reproducible search strings and processing are supplied in S1.

Item	Topic	Status	Where reported	Completion note
9	Selection of sources of evidence	Reported	Section 2.4, page 3	Screening/full-text workflow, duplicate-publication checks, single-investigator process, consensus handling of uncertain decisions, and application of the same eligibility assessment to supplementary-route reports are described.
10	Data charting process	Reported	Section 2.5, page 3	Standardized patient-level charting by one investigator, multiple-response procedure coding, consensus handling of ambiguous descriptions, and the task-limited role of GenAI are described. Final eligibility decisions and patient-level charting/extraction remained author responsibilities.
11	Data items	Reported	Section 2.5; Supplementary Table S3	Charted variables include demographics, procedure, anatomical distribution, mechanisms, symptoms, imaging, antibiotics, complications, follow-up, resolution time, and persistent outcomes. S3 also provides an audit of the clinical-variable and CT counts used in the figures.
12	Critical appraisal of individual sources (optional)	N/A - not performed	Section 2.7, page 3	No formal risk-of-bias/critical-appraisal assessment was undertaken; the rationale is reported.
13	Synthesis of results	Reported	Section 2.7, page 3; Supplementary Table S3	Descriptive synthesis, denominators, multiple-response handling, episode-level resolution analysis, and the no-meta-analysis rationale are specified. The 77-episode resolution distribution and summary statistics are reproduced in S3.
14	Selection of sources of evidence - results	Reported	Section 3.1; Figure 1; Supplementary Tables S1, S2, and S4	Record counts, screening, retrieval, exclusions, database-derived inclusions, and the source and eligibility assessment of the eight supplementary-route reports are reported.
15	Characteristics of sources of evidence	Reported	Section 3.2; Supplementary Tables S2-S3	Study-level inclusion list and patient-level characteristics are provided.
16	Critical appraisal within sources (optional)	N/A - not performed	Section 2.7	No formal critical appraisal was undertaken, so no appraisal results are applicable.

Item	Topic	Status	Where reported	Completion note
17	Results of individual sources of evidence	Reported	Supplementary Tables S2-S3	Study-level and patient-level charted data are supplied for the included evidence.
18	Synthesis of results - findings	Reported	Sections 3.2-3.6; Figures 2-7; Supplementary Table S3	Descriptive findings are synthesized across procedures, anatomy, clinical findings, mechanisms, investigations, management, outcomes, and resolution time. Multiple-response procedure totals, clinical-variable counts, CT use, and resolution statistics are auditable in S3.
19	Summary of evidence	Reported	Section 4.1 and Sections 4.2-4.7	Principal findings, relationship to prior reviews, clinical meaning, and limitations of inference are summarized.
20	Limitations	Reported	Section 4.8, page 10	Case-report bias, underreporting, missing/inconsistent data, English-language/date limits, unretrieved reports, author-developed groupings, and single-investigator screening/charting are discussed.
21	Conclusions	Reported	Section 5, page 11	Conclusions are tied to review objectives and explicitly avoid incidence/risk/treatment-effect estimates.
22	Funding	Reported	Funding statement, page 11	Funding for the scoping review is reported (“no external funding”). Funding of individual included reports was not systematically charted in the available dataset.

Important reporting note: PRISMA-ScR completion does not change the underlying review workflow. Screening and patient-level charting were performed by one investigator with coauthor discussion of uncertain cases; the manuscript reports this transparently as a limitation. Generative AI did not make final eligibility decisions or independently determine patient-level data.
